# Community health workers in Lesotho: Experiences of health promotion activities

**DOI:** 10.4102/phcfm.v10i1.1558

**Published:** 2018-02-27

**Authors:** Thato Seutloali, Lizeka Napoles, Nomonde Bam

**Affiliations:** 1 Faculty of Health Sciences, School of Health Systems and Public Health, University of Pretoria, South Africa

## Abstract

**Background:**

Lesotho adopted primary health care in 1979, and community health workers (CHWs) were included in the programme to focus on health promotion, particularly to reach people in underserved rural areas. Although the CHW programme has been successful, the heavy burden of disease because of HIV and/or AIDS and tuberculosis shifted resources from health promotion to home-based care.

**Aim:**

The study explored the lived experience of CHWs in conducting health promotion activities in Lesotho.

**Setting:**

The study was conducted in four health centres in Berea district, Lesotho.

**Methods:**

A qualitative study was conducted using an interviewer guide translated from English into Sesotho for four CHW focus group discussions, four individual interviews of key informants and four semi-structured interviews with the health centre nurses.

**Results:**

The roles of CHWs in health promotion ranged from offering basic first aid and home-based care to increasing access to health care services by taking patients to the facilities and promoting behaviour change through health education. Their perceived successes included increased access to health care services and reduced mortality rates. CHW challenges involved their demotivation to carry out their work because of lack of or inconsistent financial incentives and supplies, work overload which compromises quality of their work and limited community involvement.

**Conclusion:**

This study concludes that CHWs are beneficial to health promotion and its various activities. They had a clear understanding of their roles and responsibilities, although they did not fully comprehend that what they were describing was, in fact, health promotion. When it came to advocacy, CHWs did not fully understand it, nor did they consider it as part of their roles, although they acknowledged its importance. Their role of increasing access to health care services by accompanying patients to the facilities has increased considerably because of changes in disease burden. This is affecting their ability to practise other health promotion activities which focus on disease prevention.

## Introduction

Community health workers (CHWs) have become progressively recognised and acknowledged as an effective and efficient intervention central to increasing community-based health services, particularly in underserved areas. This mainly resulted from the 1978 Alma-Ata Declaration which identified CHWs as essential to primary health care (PHC) to attain its key target of addressing unequal and inadequate health care. This was to be achieved, among others, through community involvement and a reinforced health care workforce, which included community workers.^1^ The term ‘community health worker’ covers a broad range of community-based health service providers ‘selected, trained and working in the communities from which they come’.^2^ Although the scope of their work may vary from one country to another, they generally share similar core roles which consist of disease prevention and early detection of ill-health, community advocacy, outreach services, home visits, assisting in accessing services through referrals and follow-ups, treatment of minor ailments by administering basic first aid and providing psycho-social support through support groups.^3,4,5^ ‘In general, the role of CHWs is to act as agents of health promotion’,^6^ and their comprehensive knowledge of their communities makes them effective agents of health promotion.^7^

According to the 1986 Ottawa Charter for Health Promotion, health promotion ‘is the process of enabling individuals and communities to increase control over their health’.^8^ It can be implemented in different settings, for example villages, schools, workplaces and hospitals or clinics. In health promotion, a setting is defined as ‘a place or social context in which people engage in daily activities, in which environmental, organizational, and personal factors interact with health and wellbeing’.^9^ Health promotion encompasses multiple strategies and actions which are interconnected to enhance health. Three broad strategies should guide the planning and implementation of programmes aimed at promoting health and these include advocacy, enabling and mediation. The programme actions must also be aligned with five main action areas: building healthy public health policy, creating supportive environments, strengthening community action, developing personal skills and reorienting health services.^8,10^

The dynamics, social determinants and factors affecting health however necessitate multiple approaches to health promotion, which are (1) the medical approach, (2) the behaviour change approach, (3) the educational approach, (4) the empowerment approach and (5) the social change approach.^9^ The role of CHWs as agents of health promotion is therefore expected to be within the conceptual framework developed by the Ottawa Charter as well as the approaches outlined above.

Historically, the first significant CHW programmes began in Brazil, Tanzania and China.^11^ From the ‘community health agents’ of Brazil to the ‘barefoot doctors’ of China, CHWs have been deployed to improve the health of their communities. However, in the 1980s and the early 1990s, this PHC approach was weakened by the lack of political commitment across countries and by the lack of technical and financial support,^11^ in addition to overall weak health systems, especially in sub-Saharan Africa. Nonetheless, interest around CHWs was renewed around 2000, after the realisation that the scaling up of CHWs programmes was one of the strategies central to achieving the Millennium Development Goals.^12,13^ In fact, many programmes have sought to deliver interventions through CHWs, thus overburdening them with competing priorities which divert their focus from their regular activities.

In Lesotho, the role of CHWs dates back to as far as 1979, when the country embraced PHC^14^ and scaled up efforts to reach people in underserved and remote areas. Even though CHW programmes have been successful in that regard, the heavy burden of disease as a result of HIV and/or AIDS and tuberculosis (TB) shifted resources from health promotion to home-based care. In Lesotho, it is estimated that 23% of Basotho aged 15–49 are HIV positive^15^; 74% of TB patients are HIV positive and each year one person in every 100 develops active TB.^16^

The World Health Organization (WHO) task-shifting proposal (2006) which included the training of CHWs towards antiretroviral therapy (ART) initiatives^17^ has broadened the role of CHWs even further. Consequently, in sub-Saharan Africa, where approximately 22.5 million adults and children are living with HIV,^18^ the role of CHWs within the region has broadened and oriented towards home-based care.^17^ While this has led to increasingly empowered CHWs, it has increased their overall workload and thereby adversely affected their performance in health promotion.^19^ It is only in areas where access to ART has increased that CHWs have been able to focus on prevention or promotion.^20^

Community health workers are faced with numerous challenges. A study conducted on community health service providers in Kenya revealed that CHW challenges included the scope of their work not being clearly defined, thus affecting implementation of services, while another study reported the challenge of unavailability of resources.^13^ Other studies reported challenges such as underfunding of health educational activities and limited community participation.^13^

Some of the strategies that address CHWs’ challenges in health promotion reside in the recognition that there is a need for more community involvement. Sensitisation of the communities about the work of the CHWs is significant and likely to contribute to the sustainability of their work.^20^ Even though availability of supplies is another challenge for CHWs, there is always a likelihood of limited resources in low-income settings. Therefore, prioritisation of resources and their distribution among CHWs is vital.^12^

The aim of the study was to explore the lived experience of CHWs in conducting health promotion activities in Lesotho.

### Objectives

This study sought to establish the roles and responsibilities of CHWs in health promotion, and explored their perceptions and understanding of health promotion, as well as their perceived successes and challenges with health promotion.

### Contribution to the field

This research will be beneficial in (1) informing programme managers on CHWs’ experiences of health promotion activities and what needs to be included in their training curriculum and supervision, (2) highlighting the successes and challenges CHWs faced daily in health promotion (3) informing policymakers on how best to support the work and management of CHWs, and sustain their roles through public policy and financing, and (4) improving the documentation of CHWs’ health promotion activities.

## Research methods and design

### Study design

The study used a phenomenology qualitative research design, which includes an in-depth analysis of lived experiences and perceptions of study participants, in order to increase knowledge and understanding about a phenomenon.^21^ According to Blignault and Ritchie,^22^ ‘it is increasingly accepted that qualitative research methods should play an integral part in researching and evaluating public health activities, especially those investigating the practice of health promotion’. Therefore, the use of this design in this study facilitated an understanding of CHWs and their health promotion practices. It is an approach that increases an understanding and knowledge of ‘what works’ in health promotion.

### Study setting

The study was conducted in four health centres in Berea district, Lesotho. This district is mainly rural and located about 40 km north of the capital, Maseru. Berea was chosen for convenience, being the district where the researcher previously worked as a district health educator for 5 years. This district has 19 health centres,^23^ with approximately over 40 CHWs per health centre. The distances among different health centres are vast and, after consultation with a statistician, 4 health centres out of 19 were considered to be enough to represent the district in this study. These health centres are within easy reach of each other and the researchers would not have to travel long distances. The diversity of these selected health facilities, however, provides a wide range of CHW views and perceptions, which might be identical to those of the other health centres.

### Study population and sampling strategy

In each of the four health centres, samples consisted of seven CHWs for the group discussions, and one in-depth interview with one CHW. Also, for each health centre, one in-depth interview was conducted with the nurse-in-charge who supervises the CHWs. The recruitment of the participants was done with the assistance of the health centre nurses-in-charge and CHWs’ supervisors. Convenience sampling was used for the selection of study participants, by selecting CHWs from the areas closest to the health centres. This method was useful in saving time, money, and ensuring ease of access.^24,25^

### Data collection

Qualitative data were collected during August 2016, using a mix of face-to-face in-depth interviews and focus group discussions until saturation was reached. This was intended to enhance triangulation.^26^ Before each interview and focus group discussion, the interviewer meticulously went through the informed consent and information leaflet with the participants, after which they signed and proceeded with focus group discussions and the interviews. Data were collected at the four health centres by the principal investigator, with the assistance of a graduate support student who had familiarity with qualitative research methods. An interview guide translated from English into Sesotho by the principal investigator was used to facilitate focus on the core topics of the study for the following:

- Four focus group discussions with CHWs (one per health centre with seven participants in each group). Each discussion lasted an average of 50 min.
- Four individual interviews with CHWs (one individual CHW per health centre). This was done for more detailed accounts of their work. Each interview lasted an average of 15 min.
- Four semi-structured interviews with health centre nurses for more information on the topic. Each interview lasted an average of 15 min.
- Notes and observations taken in CHWs’ monthly meetings at two of the health centres. The meetings lasted on average 3 h.

### Data analysis

For data analysis, each focus group discussion and in-depth interview was recorded using an audio recorder, after which the recordings were transcribed word-for-word, in conjunction with translating them from Sesotho to English. These data, together with data from field notes, were analysed through thematic content analysis. The process started with identification of core topics, which have been used as themes: (1) CHWs’ own description of their core roles and responsibilities in health promotion, (2) CHWs’ understanding of health promotion and (3) successes and challenges experienced in their work. The sub-themes and categories were derived from the participants’ most common and frequent terms and words used to answer to the interview questions.

### Ethical considerations

Permission to conduct the study was obtained from the Research Ethics Committee of the University of Pretoria (Reference number: 228/2016), as well as the Research Ethics Committee of the Ministry of Health, Lesotho (Reference number: ID100-2016). Consent was obtained from the Berea District Health Management Team and from the four health centres. Consent was also obtained from the participants: before each interview and focus group discussion, the interviewer went through the informed consent and information leaflet with the participants, after which they signed it before proceeding into the interviews.

## Results

### Theme 1: Community health workers’ roles and responsibilities in health promotion

As evident from Table 1, CHWs cover a wide spectrum of activities from each of the five approaches to health promotion.

**TABLE 1:** Community health workers’ roles and responsibilities.

Health promotion approaches	Activities
**Medical approach**	Administer basic first aid Case findings and referrals Measure blood pressures, weights and heights Administer family planning pills
**Behaviour change approach**	Distributing condoms to promote practice of safe sex
**Educational approach**	Door-to-door education Health education in public gatherings and schools
**Empowerment approach**	Increasing access to food through demonstration of construction of key hole gardens Establishing support groups for ART patients, to help each other with the collection of medication refills from the facility Door-to-door education Health education in public gatherings and schools
**Social change approach**	Lead support groups Advocacy for alleviation of poverty and lack of water

ART, antiretroviral therapy.

Most of the activities identified seemed mainly home-based care related and included CHWs offering basic first aid to the people in their communities, increasing access to health care services by accompanying clients to health facilities and conducting home visits to help patients in the management of chronic illnesses, such as HIV and/or AIDS and high blood pressure. They also identified their role of providing social support through leading the establishment of support groups such as Community HIV and/or AIDS Group (CAG), a support group for ART patients, to help each other with the collection of medication refills from the facility, to prevent overcrowding at the facilities during refill collection days.

### Theme 2: Community health workers’ understanding of health promotion

**FIGURE 1:**
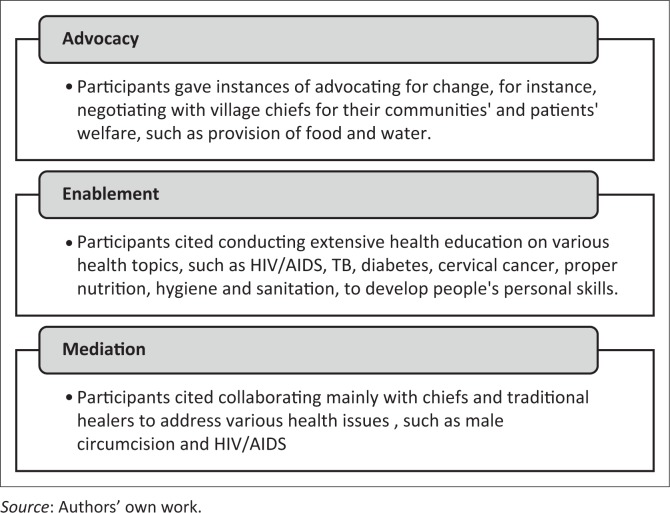
Community health workers’ understanding of health promotion.

As the approaches discussed above can be facilitated using the three main strategies of health promotion – advocacy, enablement and mediation – the participants’ understanding of what health promotion is was categorised into those strategies as presented in Figure 1.

The CHWs perceived health promotion as mainly health education, and cited conducting extensive health education on various health topics, in order to develop people’s personal skills. When it came to advocacy, they gave an example of approaching community leaders to request provision of food for patients who lack food to eat and adequate water for drinking and bathing. Furthermore, they expressed their reluctance to collaborate with politicians in issues regarding health, citing a disinclination to be identified as affiliated with political parties.

### Theme 3: Perceived successes and challenges in health promotion

**Figure 2:**
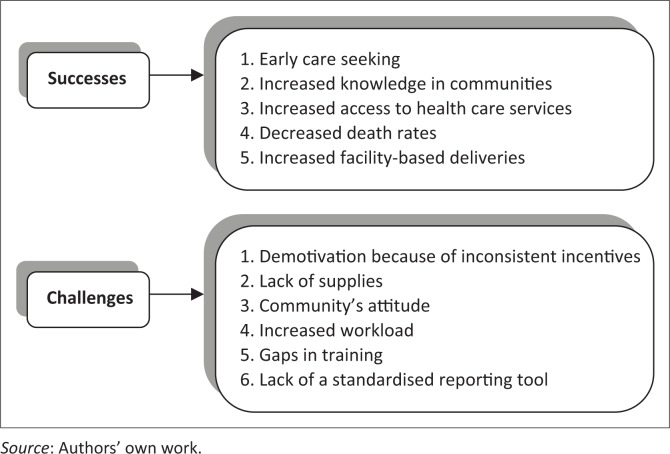
Perceived successes and challenges in health promotion.

The findings under this theme were varied but pointed to the same successes and challenges, as presented in Figure 2.

### Perceived successes in health promotion

The CHWs attribute the above perceived successes and positive changes to their health promotion activities, which are inclusive of increased knowledge of health topics in the communities, increased access to health care services, as well as early care seeking, which have led to the recovery of many patients who were on the verge of losing their lives, mainly to TB and HIV:

‘Many people could have died if it wasn’t for us.’ (Participant, female CHW, Focus Group 2)

Some of the participants cited their usefulness to the facilities, from tracking treatment defaulters and assisting patients in adherence to treatment, to lending a hand at the facilities. They claimed to have helped in the increase of facility-based deliveries by discouraging pregnant women from delivering at home where they are unable to get skilled assistance, in order to reduce maternal mortality.

‘The number of pregnant women delivering at home has greatly reduced. Right now there is nobody delivering at home. It is very rare to find such cases.’ (Participant, female CHW, Focus Group 1)

### Perceived challenges affecting practice of health promotion

#### Demotivation because of inconsistent incentives

Community health workers cited lack of or inconsistent financial incentives as a main challenge, demotivating them from carrying out their work, particularly given that they need money for transportation when they accompany patients to the facilities. They indicated that this would be the second best thing to being permanently employed by the Ministry of Health.

‘The challenge we face in our work is the issue of not receiving any incentive. Because the major concerns in a person’s life is soap to wash and food to eat … in addition to washing and eating, is having shoes on one’s feet, because one cannot come here barefooted; especially because some of us come from far, so we have to take public transport to come here. We don’t have money for transport.’ (Participant, female CHW, Individual CHW interview 4)

This challenge seemed to be echoed by the nurses interviewed. According to them, this inconsistency has affected their working relations with CHWs and the quality of their work.

#### Increased workload

The combination of their regular activities in the communities, their responsibility to track defaulters and the recent incentive-based task of accompanying people to the health facilities has increased their workload:

‘I just want to give you a scenario…you’ll find that Monday is the day for drawing blood for tests, it is a day for pregnant women who are coming here for the first time. … you come with them to the facility … and go back with them, on foot! … you’ll bring the one who is doing blood tests on Monday, the following day on Tuesday, you are bringing in one who is coming to collect their medication refills, and you come here with them. On Wednesday comes another person. … one spends the entire week bringing people to the clinic, leaving behind personal matters and chances of getting odd jobs.’ (Participant, female CHW, Individual CHW interview 4)

The nurses interviewed also reiterated these sentiments, saying that as a result of this incentive-based task of personally accompanying patients to the facilities, CHWs do not have enough time to do other health promotion activities in their communities.

#### Community’s attitude towards community health workers

The participants also mentioned that their work in health promotion has been greatly affected by the communities’ attitude towards them and lack of trust. Firstly, they claimed that CHWs lack confidentiality:

‘Some refuse to come here for treatment because they say that helps us to gain money through them. They say that we persistently come with them to the facility because that’s how we get money; they insist that nobody would do what we do if they weren’t getting paid.’ (Participant, female CHW, Individual CHW interview 4)

Secondly, the communities tended to delegate all responsibility of caring for their families to the CHWs:

‘The communities make our job harder and heavier because when they know that one is a CHW who helps patients to take their medication, they become too relaxed, in terms of not wanting to do anything to help patients, saying “Oh, so and so is coming to help”. They do not want to give their patient the medication because they expect the CHW to come and do it … they no longer even want to see to it that the patient gets something to eat.’ (Participant, female CHW, Individual CHW interview 1)

#### Lack of supplies

Participants also highlighted that they were unable to render services to their communities because of lack of supplies, such as home-based care kits, gloves and other first-aid supplies.

‘We don’t have the necessary supplies to assist the patients back home, such as gloves. That is a great challenge because when the patient has open wounds, his/her family members come to us as CHWs to ask for gloves, which we don’t have.’ (Participant, female CHW, Individual CHW interview 3)

#### Gaps in training

The participants mentioned some gaps in their training, which included few and far between refresher trainings. However, when they do get refresher trainings, they cover a wide variety of health topics, including health promotion, basic first aid and management of chronic illnesses. The most common types of training are refresher and on-the-job trainings, conducted mainly by the Ministry of Health personnel, the health centre nurses and non-governmental organisations which are in partnership with the Ministry of Health. One nurse noted:

‘They are trained on overall patient care, and on how to report, and on how to work in partnership with the facility, the villages and the chiefs and their communities.’ (Participant, female nurse, Individual Nurse interview 3)

Another gap cited by the participants was a challenge they have experienced as a result of the vertical nature of programmes. They receive training on one or other and yet communities expect them to render a comprehensive service. Nurses also expressed concern about this; they believe it has led to the deterioration in the quality of their work and has created divisions among the CHWs.

#### Lack of a standardised reporting tool for health promotion activities

While the participants seemed content with their supervision, which is done by their appointed supervisors, who are CHWs themselves, challenges remained regarding the lack of a standardised reporting tool. One nurse said:

‘There really isn’t any standardised reporting book given to them. They are just verbally informed on a format to use to report, and what to report on.’ (Participant, female nurse, Individual Nurse interview 3)

## Discussion

### Community health workers’ roles and responsibilities in health promotion

The results of this study show that health promotion roles and responsibilities of CHWs vary widely, ranging from growth monitoring and promotion to community advocacy. These findings are consistent with those reported by O’Brien et al.^3^ on the role development of CHWs. They found that the CHWs’ perceptions of their role in health promotion included providing basic first aid, conducting home visits, increasing access to health care services, promoting compliance to treatment, community outreach and providing social support.^3^ These roles and responsibilities mirror those identified by the participants in our study. This signifies a common understanding of what their core roles and responsibilities involve, a noteworthy observation because ‘in the context of CHWs, an unclear role definition may compromise the quality of patient care, resulting in poor outcomes and wasted programmatic expenditure’.^3^

While CHWs seemed to understand and clearly describe what their roles and responsibilities were, their disinclination to collaborate with politicians concerning health issues demonstrates their lack of understanding regarding the value of such collaboration in advocacy. A study by Brownstein et al. has identified advocacy as necessary for encouraging the political will to support CHW programmes,^27^ and health issues in general.

The health issues mentioned as areas of focus during the interviews are similar to those identified in the literature. These include maternal and child health, communicable and non-communicable diseases and cervical cancer.^3^ The participants further cited HIV and/or AIDS and TB as the most common health topics of their focus. This is not surprising because of the severe burden of HIV and/or AIDS and TB in Lesotho.^15,16^

### Benefits of community health workers in health promotion

The CHWs perceive their work in health promotion as beneficial for the communities they serve as well as the health systems; they reach people in their communities, who would otherwise be missed by the health care system. Even their own perceived successes are generally in agreement with the successes of CHWs as identified in reviewed literature. Wadler et al.^28^, in their study on the role of CHWs in improving breast cancer control, observed that ‘patients who work with CHWs are more likely to adhere to follow-up treatment because they have a better understanding of the health system and the course of their treatment’. They further noted that the enhanced understanding saves clinical time and resources and can lead to better outcomes for patients.^28^ For example, when CHWs perform screenings such as taking children’s weights and heights for growth monitoring, or when they support patients in adhering to treatment, facility nurses are able to focus on more technical tasks. It is, however, not clear whether they understand that growth monitoring can only be useful if accompanied by an understanding of what it means and an appropriate follow-up action or intervention.

### Community health workers’ challenges in health promotion

The challenges identified by the participants do not only affect the quality of their work and their working relationship with the nurses, but they also leave them demotivated. Consequently, when they are not addressed, they pose a threat of a significant drop out of CHWs.^19^

#### Remuneration

The primary challenge which has a bearing on their overall performance is that of inconsistent incentives. The CHWs are not necessarily challenged by their voluntarism or the amount of the recommended incentives they are supposed to receive, but they cite the need to regularise these incentives. This is a factor that should be taken into account by programme managers and non-governmental organisations when recruiting CHWs and planning sustained financing for their programme. When the issue of incentives is not addressed, it leads to CHWs seeking jobs elsewhere, thus leading to a high attrition rate. According to a study by Haines et al., this ‘contributes to decreased stability of the programme, increases training costs because of the need for continuous replacement, and makes the programme difficult to manage’.^12^

#### Increased workload

Findings also reported CHWs’ role of increasing access to health care facilities by accompanying clients to the health centres, for example, patients and/or pregnant women. Although this role seemed important to CHWs, the combination of their regular activities in the communities, coupled with this task of accompanying people to the health facilities, effectively increased their workload. This also detracted from their key performance areas of health promotion, disease prevention, early detection of illnesses and treatment adherence in the homes and communities. While the rationale for this was to ensure that people access services, it led to criticism from both the CHWs themselves and nurses as it affected their performance in provision of services at the community level. This is consistent with the findings of a study on CHWs activities conducted in Malawi, which identified that ‘increased time spent in health centres results in neglect of community-based prevention tasks’.^29^ It also confirms that increased workload for CHWs produces reduction in their performance, as identified by Prasad and Muraleedharan in their review of concepts and practices of CHWs.^19^

#### Community participation

Another factor that affects CHWs and their work is the communities which they serve. Lack of proper understanding of CHW roles and responsibilities can lead to misconceptions about their motives when they do their work. This is consistent with the findings of a study by Haines et al.^12^ which states that it is important that community awareness is raised regarding CHWs and their role in health. After all, CHWs are community-oriented^17^; therefore, ongoing support by the communities would increase the sustainability of their work.^28^

#### Supplies

It is significant to note that CHWs are also confronted by the challenge of lack of supplies and equipment they need to use in their work, such as gloves, bandages, weighing scales for growth monitoring and general home-based care kits. Adequate supplies can enhance their performance and motivation to do their work. While limited resources are likely to be always a challenge in low-income settings, prioritisation of resources and their distribution among CHWs is vital.^13^

#### Gaps in training

The study highlights the challenges posed by the vertical nature of the prioritised health programmes. This leads to programme-specific training and this presents challenges with implementing an integrated package in the communities. Even though this approach may be beneficial, moving from the generalist CHW to a more focused role, a study by Wadler et al. has shown that CHWs function most effectively in a generalised way than a programme-specific one.^28^ The whole approach to household and community interventions is integrated and comprehensive care.

#### Standardised reporting tool

To monitor the efforts of CHWs in health promotion, it is important to have a standardised reporting tool for their health promotion activities. As CHWs serve diverse populations, address various health issues and use multiple approaches in their work, it is important to identify common deliverables or outcomes expected from their work and develop a tool based on that, which would be useful in collecting and analysing data, and reporting. The significance of such a tool is that their reports can be used for future research purposes and improvement of the programme.

### Further research

To ensure the sustainability of the CHWs programme, further research is necessary to consider the best methods for monitoring and evaluating their health promotion activities and quality of their work, as well as to evaluate the best methods for their training in health promotion. More research on CHWs has ‘the potential to increase funding for CHW programmes and recognition of CHWs as essential members of the health care workforce’.^4^ There is further research necessary on the issue of specialisation of CHWs as opposed to a more broad approach to their work.^29^

### Limitations of the study

This study has several limitations. Firstly, it was conducted in one district out of 10; long distances between districts would have time and cost implications for the researcher. The diversity of the health facilities selected, however, provides a wide range of CHW views and perceptions, which might be identical to those of other districts. In addition, the fact that the researcher had previously worked in the district and with some of the study participants may have resulted in interviewer and interviewee bias.

### Recommendations

- Sensitisation and community awareness regarding the role of CHWs in promoting individual, family and community health outcomes so as to change their attitudes towards CHWs.
- Development of strategies to review the scope of work so as to reduce their workload while maintaining effectiveness and good quality standards.
- Review of employment of CHWs, their remuneration and budgets by health care authorities and non-government organisations.
- Review sustainable and cost-effective means of supplying CHWs with home-based care kits, equipment and supplies to enhance their work.
- Strengthening of the capacity of CHWs through frequent refresher trainings, work-related onsite training and regular competency assessments.
- Development of clear monitoring and evaluation tools for health promotion activities based on defined performance standards.
- Improvement of data recording and reporting tools, coupled with data utilisation and response to issues identified at the local level.

## Conclusion

In conclusion, this study shows that the CHWs in Lesotho have an adequate understanding of their roles and responsibilities regarding health promotion. However, the changes in disease burden have resulted in a shift in roles and this is affecting their health promotion practice and experience. In addition, the frequent, newly emerging and re-emerging health issues are likely to keep evolving and broadening their health promotion activities.

The findings of this study make a valuable contribution to public health by generating needed and necessary evidence to improve CHW programmes. The analysis in the study has made an effort to fill the gaps in existing literature on CHWs and their health promotion activities. It is therefore essential to keep developing strategies to reduce their workload, while maintaining their primary roles and the quality of their work. Hence, governments and non-governmental organisations should work hand-in-hand to address their employment status, funding and support.

## References

[1] World Health Organization Declaration of Alma-Ata: International Conference on Primary Health Care [homepage on the Internet]. Vol. 6 Alma-Ata; 1978 [cited February 2017]. Available from: http://www.who.int/publications/almaata_declaration_en.pdf

[2] Hermann K, Van Damme W, Pariyo GW, et al. Community health workers for ART in sub-Saharan Africa: Learning from experience–capitalizing on new opportunities. Hum Resour Health. 2009;7:31 https://doi.org/10.1186/1478-4491–7–31 19358701 10.1186/1478-4491-7-31 PMC2672918

[3] O’Brien MJ, Squires AP, Bixby RA, Larson SC Role development of community health workers: An examination of selection and training processes in the intervention literature. Am J Prev Med. 2009;37(6):S262–S269. https://doi.org/10.1016/j.amepre.2009.08.011 19896028 10.1016/j.amepre.2009.08.011 PMC2856599

[4] Rosenthal EL, Wiggins N, Ingram M, Mayfield-Johnson S, Zapien JGD Community health workers then and now: An overview of national studies aimed at defining the field. J Ambul Care Manage. 2011;34(3):247–259. https://doi.org/10.1097/JAC.0b013e31821c64d7 21673523 10.1097/JAC.0b013e31821c64d7

[5] Ingram M, Reinschmidt KM, Schachter KA, et al. Establishing a professional profile of community health workers: Results from a national study of roles, activities and training. J Community Health. 2012;37(2):529–537. https://doi.org/10.1007/s10900-011-9475-2 21964912 10.1007/s10900-011-9475-2 PMC6684283

[6] Lehmann U, Friedman I, Sanders D Review of the utilisation and effectiveness of community-based health workers in Africa JLI Working Paper. South Africa: Global Health Trust, Joint Learning Initiative on Human Resources for Health and Development (JLI), 2004; p. 4–1. Cape Town, South Africa.

[7] Kash BA, May ML, Tai-Seale M Community health worker training and certification programs in the United States: Findings from a national survey. Health Policy. 2007;80(1):32–42. https://doi.org/10.1016/j.healthpol.2006.02.010 16569457 10.1016/j.healthpol.2006.02.010

[8] World Health Organization Milestones in health promotion: Statements from global conferences. Vol. 9(1). WHO/NMH/CHP/0901 Geneva, Switzerland: World Health Organization; 2009.

[9] Moodie R, Hulme A, editors Hands-on health promotion. Melbourne: IP Communications Pty. Ltd.; 2004, p. 418.

[10] World Health Organization The Bangkok Charter for Health Promotion in a globalized world. Health Promot Int. 2006;21(Suppl 1):10–14. https://doi.org/10.1093/heapro/dal046 17307952 10.1093/heapro/dal046

[11] Liu A, Sullivan S, Khan M, Sachs S, Singh P Community health workers in global health: Scale and scalability. Mt Sinai J Med. 2011;78(3):419–435. https://doi.org/10.1002/msj.20260 21598268 10.1002/msj.20260

[12] Haines A, Sanders D, Lehmann U, et al. Achieving child survival goals: Potential contribution of community health workers. Lancet. 2007;369(9579):2121–2131. https://doi.org/10.1016/S0140-6736(07)60325-0 17586307 10.1016/S0140-6736(07)60325-0

[13] Oliver M, Geniets A, Winters N, Rega I, Mbae SM What do community health workers have to say about their work, and how can this inform improved programme design? A case study with CHWs within Kenya. Glob Health Action. 2015;8:27168. 26004292 10.3402/gha.v8.27168 PMC4442123

[14] Ministry of Health and Social Welfare Lesotho PHC revitalisation plan: 2011–2017 [homepage on the Internet]. Maseru: Ministry of Health and Social Welfare (MOHSW); 2010 [cited 2017 Oct]. Available from: http://www.nationalplanningcycles.org/sites/default/files/country_docs/Lesotho/lesotho_phc_action_plan_2011_2017_draft_submitted_to_moh_3_2_5.pdf

[15] Ministry of Health and Social Welfare, ICF Macro Lesotho demographic and health survey 2009 [homepage on the Internet]. Maseru: MOHSW and ICF Macro; 2010, 452 p [cited 2017 Feb]. Available from: https://dhsprogram.com/pubs/pdf/FR241/FR241.pdf

[16] World Health Organization Global tuberculosis report 2014. Geneva: World Health Organization; 2014.

[17] Schneider H, Hlophe H, Van Rensburg D Community health workers and the response to HIV/AIDS in South Africa: Tensions and prospects. Health Policy Plan. 2008;23(3):179–187. 18388133 10.1093/heapol/czn006

[18] World Health Organization HIV in the WHO African region: Progress towards achieving universal access to priority health sector interventions. Geneva: World Health Organization; 2011.

[19] Prasad BM, Muraleedharan VR Community health workers: A review of concepts, practice and policy concerns [homepage on the Internet]. A review as part of ongoing research of International Consortium for Research on Equitable Health Systems (CREHS); 2007 [cited 2017 Feb]. Available from: https://pdfs.semanticscholar.org/2c61/fe3a28a53087aee94583b6f669b3a4b4825d.pdf

[20] Dennill K, Rendall-Mkosi K Primary health care in Southern Africa: A comprehensive approach. 3rd ed. Cape Town: Oxford University Press Southern Africa (Pty) Ltd; 2012, p. 256.

[21] Starks H, Trinidad SB Choose your method: A comparison of phenomenology, discourse analysis, and grounded theory. Qual Health Res. 2007;17(10):1372–1380. https://doi.org/10.1177/1049732307307031 18000076 10.1177/1049732307307031

[22] Blignault I, Ritchie J Revealing the wood and the trees: Reporting qualitative research. Health Promot J Aust. 2009;20(2):140–145. 10.1071/he09140 19642963

[23] Mwase T, Kariisa E, Doherty J, Hoohlo-Khotle N, Kiwanuka-Mukiibi P, Williamson T Lesotho health systems assessment 2010. Volume 20 Bethesda, MD: Health Systems; 2010, p. 20.

[24] Creswell JW Qualitative inquiry and research design: Choosing among five approaches. Thousand Oaks, CA: Sage; 2007.

[25] Miles MB, Huberman AM, Saldana J Qualitative data analysis: A methods sourcebook. Thousand Oaks, CA: Sage; 2013.

[26] Mouton J, Babbie E The practice of social research Cape Town: Wadsworth Publishing Company; 2001.

[27] Brownstein JN, Bone LR, Dennison CR, Hill MN, Kim MT, Levine DM Community health workers as interventionists in the prevention and control of heart disease and stroke. Am J Prev Med. 2005;29(5 Suppl 1):128–133. https://doi.org/10.1016/j.amepre.2005.07.024 16389138 10.1016/j.amepre.2005.07.024

[28] Wadler BM, Judge CM, Prout M, Allen JD, Geller AC Improving breast cancer control via the use of community health workers in South Africa: A critical review. J Oncol. 2011;2011 https://doi.org/10.1155/2011/150423 10.1155/2011/150423 PMC2948888 20936151

[29] Smith S, Deveridge A, Berman J, Negin J, et al. Task-shifting and prioritization: A situational analysis examining the role and experiences of community health workers in Malawi. Hum Resour Health. 2014;12(1):24. https://doi.org/10.1186/1478-4491-12-24 10.1186/1478-4491-12-24 PMC4014628 24885454

